# *Staphylococcus aureus* enhances biofilm formation, aerotolerance, and survival of *Campylobacter* strains isolated from retail meats

**DOI:** 10.1038/s41598-021-91743-w

**Published:** 2021-07-05

**Authors:** Anand B. Karki, Kaylee Ballard, Claudia Harper, Robert J. Sheaff, Mohamed K. Fakhr

**Affiliations:** 1 Department of Biological Science, The University of Tulsa, Tulsa, OK USA; 2 Department of Chemistry and Biochemistry, The University of Tulsa, Tulsa, OK USA

**Keywords:** Microbiology, Bacteriology

## Abstract

In retail meat products, *Campylobacter jejuni*, *C. coli*, and *Staphylococcus aureus* have been reported in high prevalence. The polymicrobial interaction between *Campylobacter* and other bacteria could enhance *Campylobacter* survival during the adverse conditions encountered during retail meat processing and storage. This study was designed to investigate the potential role of *S. aureus* from retail meats in enhancing the survival of *Campylobacter* exposed to low temperature, aerobic conditions, and biofilm formation. Results indicated that viable *S. aureus* cells and filter-sterilized cell-free media obtained from *S. aureus* prolonged the survival of *Campylobacter* at low temperature and during aerobic conditions. Biofilm formation of *Campylobacter* strains was significantly enhanced in the presence of viable *S. aureus* cells, but the results were inconclusive when extracts from cell-free media were used. In conclusion, the presence of *S. aureus* cells enhances survivability of *Campylobacter* strains in adverse conditions such as low temperature and aerobic conditions. Further investigations are warranted to understand the interaction between *Campylobacter* and *S. aureus*, and effective intervention strategies are needed to reduce the incidence of both foodborne pathogens in retail meat products.

## Introduction

The high prevalence of *Campylobacter* spp. and *Staphylococcus aureus* in retail meat products^1–4^ remains a challenge for food safety despite the improvement in retail meat production systems. Sporadic outbreaks of campylobacteriosis and staphylococcal food poisoning after consumption of contaminated food products has been reported worldwide^5–8^. A recent report from the CDC has documented an incremental increase in campylobacteriosis cases in the USA in recent years^9^. *Campylobacter* is a microaerobic, fastidious organism that lacks genetic mechanisms to cope with various stressors^10–12^; however, it persists in food products even with exposure to harsh environmental conditions during processing and storage^1,2,13^. Temperature fluctuations beyond its optimal growth temperature (37–42 °C) and exposure to aerobic conditions (oxidative stress) are major stressors that *Campylobacter* encounters during food processing and storage^14,15^. Survival mechanisms of *Campylobacter* include the viable but non-culturable condition (VBNC), biofilm formation, aerotolerance, and acquisition of cryoprotectant molecules^14,15^. Biofilm production by foodborne pathogens in retail meat processing surfaces and environments facilitates their survival^16,17^ and ensures the contamination of retail meat products. In situations where enhanced survival and biofilm formation have been reported in retail meat and liver products, the food matrix environment influenced the survival of *Campylobacter*^18,19^. Meanwhile, the higher prevalence of aerotolerant *Campylobacter* strains from food products also ensures their survival during oxidative stress^20,21^.

The high prevalence of *S. aureus* as a co-contaminant with *Campylobacter* in the same retail meat source has been previously reported by our laboratory^1–4,13,22^. Meanwhile, many other co-contaminants, including *Pseudomonas* spp., *Salmonella* spp*., Staphylococcus* spp*.*, hepatitis E, *Escherichia* spp. and *Yersinia* spp. are also prevalent in retail meat and liver products^23–25^. Likewise, many spoilage bacteria have been identified in biofilms in meat processing environments that are known to contaminate retail meat products^16,17^. The presence of other co-contaminants could enhance survival of *Campylobacter* spp. during adverse environmental conditions^26,27^, which would ensure its persistence in the retail meat environment. *Campylobacter* has been characterized as a secondary colonizer in pre-existing biofilms^26^. However, there are very few studies documenting higher biofilm formation and enhanced survival of *Campylobacter* in adverse environments that include polymicrobial interactions^26–30^.

*Staphylococcus aureus* can grow aerobically within a wide range of temperatures^31^; consequently, it might provide a better environment for survival of co-contaminant *Campylobacter* during food processing and storage. A few reports exist documenting the coexistence of *C. jejuni* and *S. aureus* in biofilms^28,29^; however, the influence of *S. aureus* strains from retail meats on the survival of *C. jejuni* and *C. coli* at low temperatures and aerobic conditions is unclear. In this study, we studied the influence of *S. aureus* strains from retail meat products on *Campylobacter* survival at low temperature, in aerobic conditions and within biofilms. Cell-free *S. aureus* were included to investigate the effect of extracellular metabolites on *Campylobacter* survival during adverse environmental conditions.

## Materials and methods

### Bacterial strains and culture conditions

Five *Campylobacter* strains, including two and three strains of *C. jejuni* and *C. coli*, respectively, and two *S. aureus* strains were sourced from retail meat products and used in this study (Table 1). These strains were previously isolated, characterized and sequenced in our laboratory^1–4,12,13,22,32–37^. These strains were selected because the genomic sequences were available, and they represent different sources of retail meats. The clinical strain *C. jejuni* NCTC11168 was used as a reference strain. *Campylobacter* strains were subcultured from stock culture (stored at − 70 °C) in Mueller Hinton Agar (MHA) supplemented with 5% laked horse blood (at 42 °C) in microaerobic conditions (Oxoid CampyGen 3.5 L sachet, Thermo Scientific or Thermo Forma tri-gas incubator) for 48 h. Subcultures of *Campylobacter* cultures were maintained on MHA plates or Mueller Hinton Broth (MHB) as needed. *S. aureus* strains were grown and subcultured in Mannitol Salt Agar (MSA) at 37 °C in aerobic conditions.

**Table 1:** Bacterial strains used in this study. All *Campylobacter* strains (except *C. jejuni* NCTC11168) and *S. aureus* strains were isolated and whole-genome sequenced in our laboratory^1–4,12,13,22,32–37^.

Bacterial strains	Source	Accession number (chromosome, plasmids)
*C. jejuni* T1-21	Chicken meat	CP013116.1, CP013117.1
*C. jejuni* OD2-67	Chicken liver	CP014744.1, CP014745.1, CP014746.1
*C. jejuni* NCTC11168	Clinical (reference strain)	Al111168.1
*C. coli* WA3-33	Chicken liver	CP017873.1, CP017874.1
*C. coli* HC2-48	Beef liver	CP013034.1, CP013035.1
*C. coli* ZV1-224	Pork meat	CP017875.1, CP017876.1, CP017877.1
*S. aureus* B4-59C	Chicken meat	CP042153.1, CP042154.1, CP042155.1, CP042156.1
*S. aureus* B6-55A	Turkey meat	CP042110.1–CP042152.1

### Preparation of cell-free extracts from *S. aureus*

*Staphylococcus aureus* strains B4-59C and B6-55A were grown in MSA for 24 h; cells were then subcultured in freshly-prepared MHB (50 ml) and incubated at 37 °C at 100 rpm for 24 h with aerobic conditions. Cell pellets were obtained after centrifugation (5000 rpm, 5 min), washed in PBS (pH 7.4), suspended in fresh MHB, and adjusted to OD_600_ = 1.0. Twenty milliliters of cell suspension was added to 200 ml MHB and incubated at 4 °C for 48 h. Similarly, 10 ml of cell suspension was added to 200 ml MHB in conical flasks (250 ml) and incubated separately at 25 °C and 37 °C with 100 rpm agitation for 24 h. After incubation, inoculated MHB media were filter-sterilized (0.45 µm, Nalgene Rapid-Flow), and sterility was verified on MHA, MHA supplemented with 5% laked horse blood and MSA incubated in aerobic and microaerobic conditions at 37 °C and ambient temperature for 48 h. For each temperature setting (4 °C, 25 °C and 37 °C), experiments were performed in triplicate; filter-sterilized media were mixed and stored at − 20 °C. These sterilized media included: *S. aureus* grown in MHB at 4 °C (strains B4-49C-4 and B6-55A-4), *S. aureus* grown in MHB at 25 °C (strains B4-59C-25 and B6-55A-25), *S. aureus* grown in MHB at 37 °C (strains B4-59C-37 and B6-55A-37), and non-inoculated MHB (control); these media were used in survival, biofilm and aerotolerance assays of *Campylobacter* strains. The protein concentration of inoculated and non-inoculated MHB was measured by the BCA protein Assay kit (Pierce™).

### Fractionation of media used to cultivate *S. aureus*

Filter-sterilized media from *S. aureus* cultures were stored at − 20 °C and thawed overnight at 4 °C prior to use. Filter-sterilized media were fractionated using a Ultracel-30 kDa filter **(**Millipore, Burlington, Massachusetts, USA), and the eluate (≤ 30 kDa) was centrifuged for 30 min at 5000 rpm. For all samples, 20 µl of non-inoculated MHB and both fractions (≤ 30 kDa and ≥ 30 kDa) were separated in 12% polyacrylamide gels by electrophoresis.

### *Campylobacter* survival assays at 4 °C

#### Survival assays using cell-free extracts from S. aureus

*Campylobacter jejuni* NCTC11168, T1-21 and OD2-67 and *C. coli* HC2-48, ZV1-224 and WA3-33 were cultured overnight on MHA supplemented with 5% laked horse blood. Cells were removed, suspended to OD_600_ = 0.1 in MHB, and then diluted tenfold in either MHB or cell-free extracts of *S. aureus* that were chilled to (4 °C). The cell-free extracts included *S. aureus* B4-59C grown at 4, 25, and 37 °C (B4-59C-4, B4-59C-25, B4-59C-37) and *S. aureus* B655A grown at 4, 25, and 37 °C (B655A-4, B6-55A-25 and B6-55A-37). Triplicate sets of *Campylobacter*-inoculated media (8 ml) were incubated at 4 °C in polystyrene culture tubes. At 0, 24, 48, 72 and 120 h, 40 µl samples were removed and serial dilutions were made in PBS (pH 7.4). Viable cell counts were obtained by spotting 10 µl dilutions on MHA and incubating in microaerobic condition (Thermo Forma tri-gas incubator) as described previously^18^.

#### Survival assays using S. aureus cells

*Campylobacter* strains were grown for 48 h in MHA supplemented with 5% laked horse blood and the following antibiotics: cefoperazone, 20 µg/ml; vancomycin, 20 µg/ml; trimethoprim, 20 µg/ml; and amphotericin B, 10 µg/ml {(Bolton Selective Supplement FD231, Himedia, Mumbai, India)}. *Campylobacter* cells were then subcultured in 25 ml MHB at 110 rpm, 42 °C with microaerobic conditions for 24 h. *S. aureus* strains were grown in MSA from stock cultures for 24 h and subcultured in 25 ml MHB for 18 h at 37 °C with aerobic conditions. *Campylobacter* and *S. aureus* cells were pelleted and resuspended in MHB to OD_600_ = 0.1. For *Campylobacter*, cell suspensions were mixed in MHB preincubated at 4 °C to create a 1:5 dilution, and this was used for both mono and mixed cultures. A 1:10 dilution of *S. aureus* cell suspension was added to *Campylobacter* cells in MHB for the mixed culture experiment. Triplicate samples (5 ml) of *Campylobacter* monocultures or mixed cultures (*S. aureus* + *Campylobacter*) were incubated at 4 °C in polystyrene culture tubes. Viable counts for *Campylobacter* were obtained on MHA amended with Bolton Selective Supplement FD231 at 42 °C in microaerobic conditions (Oxoid CampyGen 3.5 L sachet, Thermo Scientific). Meanwhile, *S. aureus* counts were obtained on MSA at 37 °C with aerobic incubation. Viable counts were recorded up to 7 days; this data point was selected because *Campylobacter* monocultures could only produce colonies on MHA (with antibiotics) up to 5 days of incubation.

### *Campylobacter* aerotolerance assays

#### Aerotolerance assays using cell-free extracts from S. aureus

Aerotolerance of *Campylobacter* strains was assessed as described previously with minor modifications^18,21^. MHB and cell-free extracts from *S. aureus* B4-59C-4, B4-59C-25, B4-59C-37, B6-55A-4, B6-55A-25, and B6-55A-37 were used in the experiment. *Campylobacter* strains were subcultured in freshly-prepared MHB and incubated to an OD_600_ = 0.5; a 500 µl aliquot was then added to 5 ml of media (MHB and *S. aureus* cell-free extracts). Triplicate sets (1.5 ml each) were transferred to 50 ml Falcon tubes with vented (cracked open) caps to simulate aerobic conditions and incubated at 42 °C, 200 rpm for 12–24 h. Samples (100 µl) were taken at 0, 6, 12, and 24 h, and viable counts were obtained on MHA (Thermo Forma tri-gas incubator). Each experiment was repeated at least twice.

#### Aerotolerance assays using S. aureus cells

*Campylobacter* and *S. aureus* strains were subcultured in 25 ml freshly-prepared MHB. Suspensions were centrifuged at 10,000 rpm for 5 min, and pellets were resuspended in MHB at OD_600_ = 0.5 for *Campylobacter* and OD_600_ = 0.1 for *S. aureus*. A tenfold dilution of each *Campylobacter* strain was prepared in 30 ml freshly-prepared MHB and divided equally into triplicate, 10 ml aliquots. One set of suspensions was used as a control and contained only *Campylobacter* cells. In another set of suspensions, mixed populations of *S. aureus* and *Campylobacter* were prepared by combining *S. aureus* B4-59C or B6-55A (1 ml, OD_600_ = 0.1) with a 10 ml suspension of *Campylobacter* cells. *Campylobacter* monocultures were prepared as described above for survival assays, and *S. aureus* monocultures were prepared for both strains by adding 1 ml of *S. aureus* cell suspension to 10 ml freshly-prepared MHB. Triplicate aliquots (1 ml) of inoculated media (*Campylobacter* and *S. aureus* mixed cultures and monocultures of the two organisms) were incubated in 24-well culture plates at 25 °C or 42 °C in aerobic conditions at 200 rpm. To ensure proper aeration, lid of 24-well culture plate was lifted higher using adhesive tapes. Viable counts for *Campylobacter* were obtained at 0, 6, 12 and 24 h in MHA supplemented with antibiotics (Bolton Selective Supplement FD231); cultures were incubated at 42 °C in microaerobic conditions (Oxoid CampyGen 3.5 L sachet, Thermo Scientific) for at least 48 h. *S. aureus* cell counts were obtained at 0, 6, 12 and 24 h on MSA plates that were incubated at 37 °C for 24 h.

### Biofilm assays

#### *Biofilm formation using cell free extracts and* < *30 kDa media fractions*

*Campylobacter* strains and cell-free extracts from *S. aureus* B4-59C-4, B4-59C-25, B4-59C-37, B655A-4, B6-55A-25 and B6-55A-37 were prepared as described above in aerotolerance and survival assays, respectively. *Campylobacter* monocultures and cells amended with *S. aureus* cell-free extracts or the ≤ 30 kDa flow-through fraction were analyzed for biofilm formation as described previously^18^. The ≥ 30 kDa fraction was excluded due to insufficient volume. Suspensions were diluted tenfold, and four replicate samples (150 µl) were incubated (static) in 96-well polystyrene flat-bottomed plates at 42 °C in microaerobic environment (Thermo Forma tri-gas incubator) or aerobic condition (normal atmospheric condition) for 72 h. The lid of 96 well plate was raised higher using adhesive tape to ensure proper ventilation as mentioned earlier when describing the aerotolerance assay. After 72 h, cell suspensions were then discarded, and plates were rinsed with demineralized water. Plates were dried at 60 °C for 30 min and crystal violet (200 µl; 1%) was added to each well; plates were then gently rocked for 30 min at ambient temperature. Plates were thoroughly washed with demineralized water and allowed to dry at 37 °C. Biofilms were solubilized by adding 180 µl of 20% acetone and 80% ethanol to each well. Bound crystal violet was measured at *A*_595_ with an Appliskan Multimode Microplate Reader (Thermo Scientific, Waltham, MA).

#### Biofilm assays with S. aureus cells

Bacterial suspensions of mono and mixed cultures were prepared as described above for aerotolerance assays with *S. aureus* cells. Four replicate aliquots (150 µl) of mono and mixed cultures were dispensed into 96-well polystyrene plates and incubated (static) for 48 h at 42 °C or 25 °C in aerobic conditions (normal atmospheric environment) as mentioned previously. After incubation, biofilms were stained with crystal violet staining as described previously^38^. Each experiment was conducted at least twice.

### Statistical analyses

Results were analyzed using Two-way ANOVA (Tukey multiple comparison test), One-way ANOVA (Brown-Forsythe and Welch ANOVA tests) and unpaired t-test as per need in GraphPad (Prism 9), and illustrations were created in GraphPad and Microsoft Excel.

## Results

### *Campylobacter* survival at low temperature

**Figure 1:**
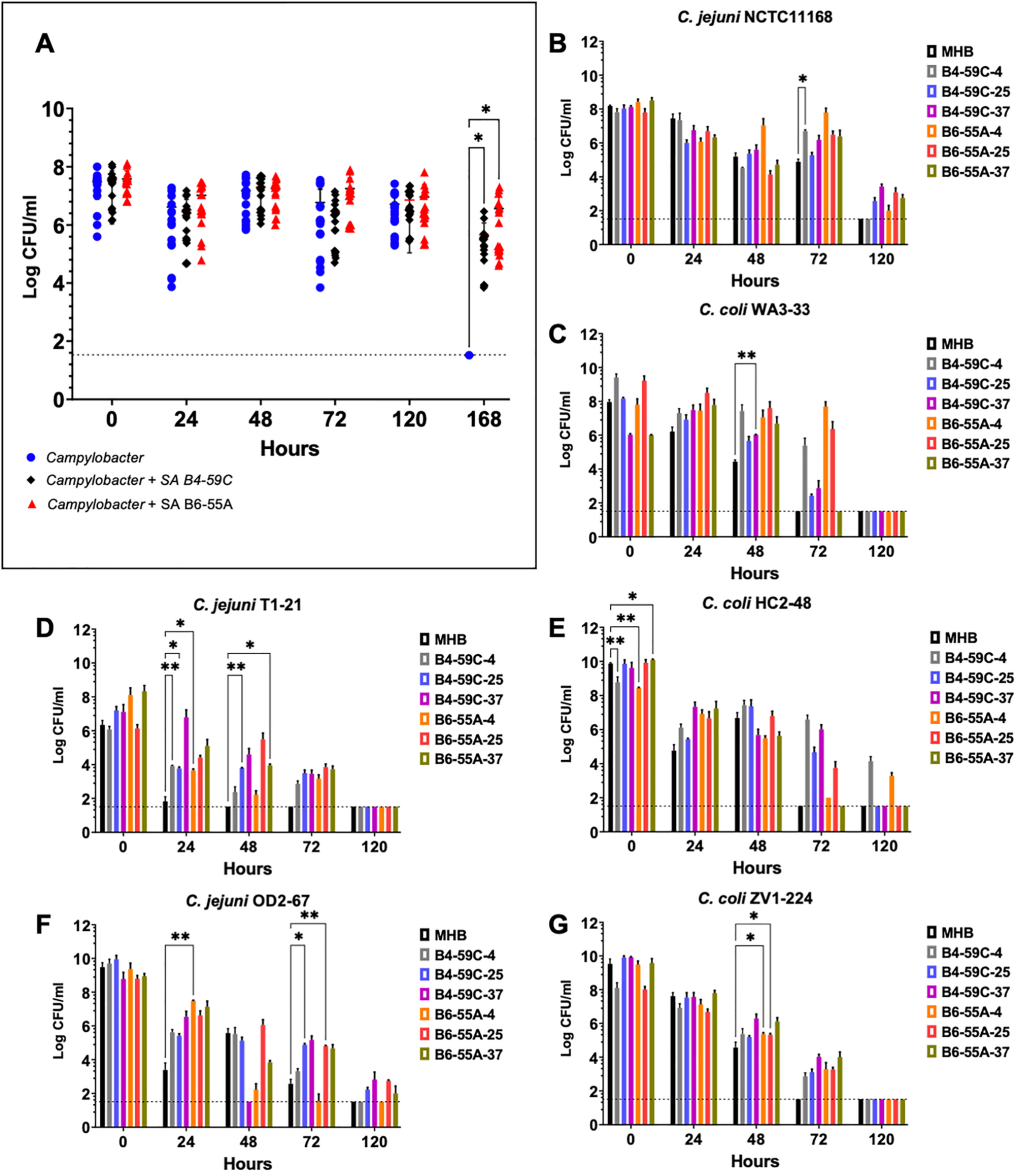
Survival of *Campylobacter* strains at low temperature (4 °C) in mono or mixed cultures containing *S. aureus* strains or cell-free extracts. (**A**) Survival of *Campylobacter* strains (all *C. jejuni* and *C. coli* strains) monocultures and mixed cultures containing *S. aureus* B4-59C or B6-55A. All individual viable count readings from *Campylobacter* monocultures or mixed cultures are represented in figure (**A**). Survival of (**B**) *C. jejuni* NCTC11168, (**C**) *C. coli* WA3-33, (**D**) *C. jejuni* T1-21, (**E**) *C. coli* HC2-48, (**F**) *C. jejuni* OD2-67 and (**G**) *C. coli* ZV1-224 in media containing cell-free extracts of *S. aureus* at 4 °C. The cell-free extracts were obtained from *S. aureus* B4-59C grown at 4, 25, and 37 °C (B4-59C-4, B4-59C-25, B4-59C-37) and *S. aureus* B6-55A grown at 4, 25, and 37 °C (B6-55A-4, B6-55A-25 and B6-55A-37). *Campylobacter* cells grown in non-amended MHB served as a control. Each bar in figures (**B**–**G**) represents the mean of colony counts with standard deviation (SD) error bar. Horizontal dotted lines in the figures represent the limit of detection for colony counts (LOD, ~ 33 CFU/ml) in these assays. All data points plotted on the LOD line in figure (**A**) and all the bars not exceeding LOD line in figures (**B**–**G**) represent the readings for the respective time points with no detectable CFU counts. Tukey multiple comparison tests (Two-way ANOVA with repeated measures) was conducted in GraphPad Prism 9: *p < 0.05, **p < 0.01, ***p < 0.001, ****p < 0.0001.

Prolonged survival of *Campylobacter* at 4 °C was found when cells were co-cultivated with *S. aureus* B6-55A or B4-59C as compared to cells consisting of *C. jejuni* and *C. coli* monoculture (Fig. 1A, Supplementary Fig. S1). Likewise, cell-free extracts from *S. aureus* B4-59C-4 and B6-55A-4 increased survival of most *Campylobacter* strains at 4 °C when added to the media as compared to the non-amended control (MHB) (Fig. 1B–G). Cell-free extracts from *S. aureus* cultured at 25 °C and 37 °C (B4-59C-25, B4-59C-37, B6-55A-25 and B6-55A-37) also enhanced prolonged survival of *C. jejuni* and *C. coli* at 4 °C as compared to MHB (Fig. 1B–G). Among most of the *Campylobacter* strains (both *C. jejuni* and *C. coli* strains), effects of growth medium (MHB vs cell free extracts from *S. aureus* cultures), time, and time X growth medium interaction were found significant on survival at lower temperature (Supplementary Table S2.1–S2.3). Significant effects of growth condition (monoculture vs polymicrobial culture), time, and interaction of time X growth condition on survival at lower temperature were found for *C. jejuni* (NCTC11168, OD2-67) and *C. coli* HC2-48 strains (Supplementary Table S2.1–S2.3). Viability of *S. aureus* cells showed a tenfold (1 log) reduction after 120 h at 4 °C (Supplementary Table S3). No significant variability was observed for survival of *S. aureus* in mixed culture with the six different strains of *Campylobacter*.

### *Campylobacter* survival during aerobic conditions

**Figure 2:**
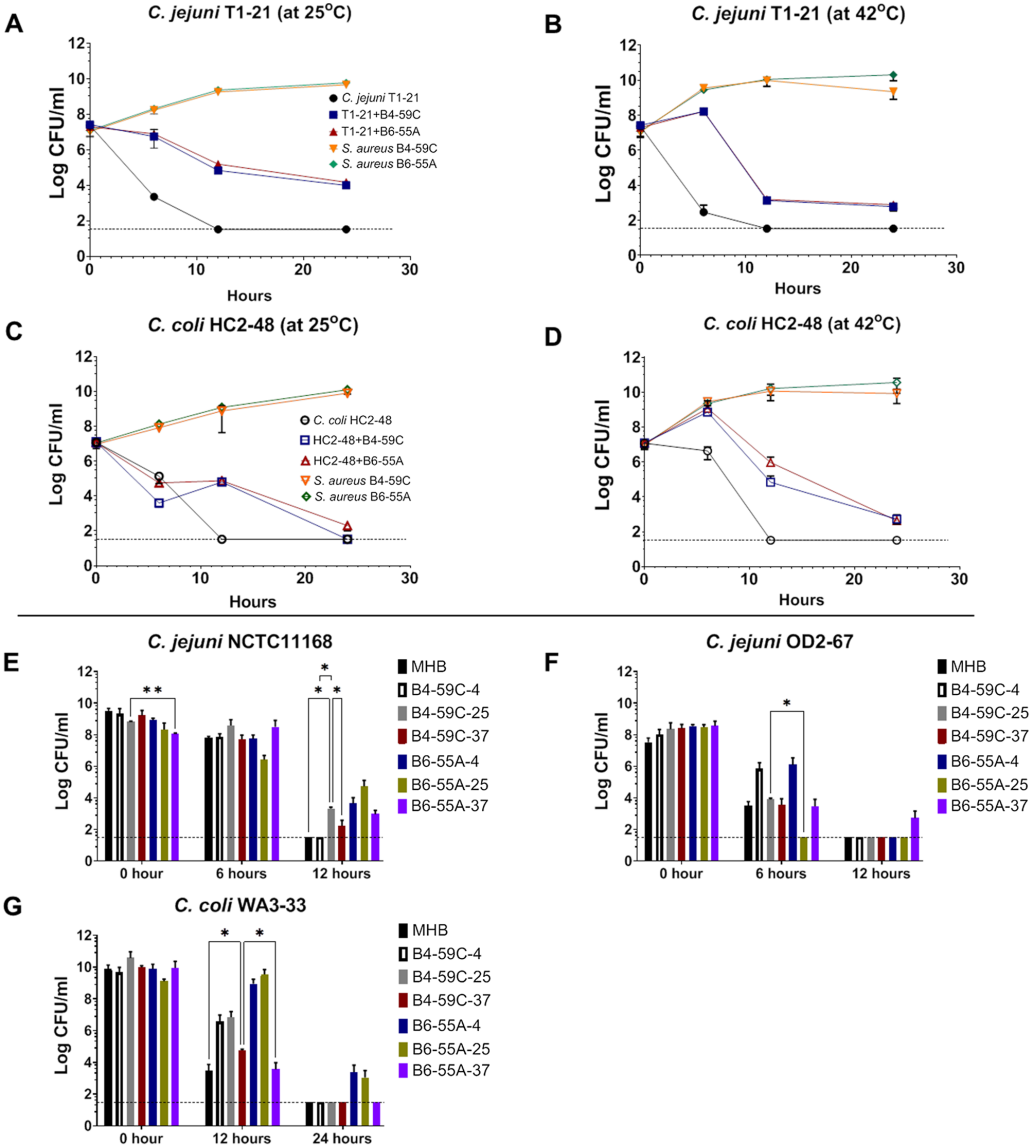
Aerotolerance of *Campylobacter* in mono or mixed cultures containing *S. aureus* strains or cell-free extracts. (**A**–**D**), Survival of *C. jejuni* T1-21 and *C. coli* HC2-48 in mono and mixed cultures of *S. aureus* B4-59C and B6-55A incubated at 25 °C and 42 °C with aerobic conditions. (**E**) Survival of *C. jejuni* NCTC11168, (**F**) *C. jejuni* OD2-67, and (**G**) *C. coli* WA3-33 in media containing cell-free extracts of *S. aureus* at 42 °C with aerobic conditions. The *S. aureus* cell-free extracts listed in the Fig. 1 legend were used in this experiment. *Campylobacter* cells grown in non-amended MHB served as a control. Mean values of CFU counts with SD error bars are represented in figures. Horizontal dotted lines in the figures represent the limit of detection for colony counts (LOD, ~ 33 CFU/ml) in these assays. All data points plotted on the LOD line in figure (**A**–**D**) and all the bars not exceeding LOD line in figures (**E**–**G**) represent the readings for the respective time points with no detectable CFU counts. Two-way ANOVA with repeated measures (Tukey multiple comparison tests) was conducted in GraphPad Prism 9: *p < 0.05, **p < 0.01, ***p < 0.001, ****p < 0.0001.

During aerobic incubation at 25 °C and 42 °C, *Campylobacter* survival was enhanced throughout the incubation period when cells were co-incubated with *S. aureus* (Fig. 2A–D, Supplementary Fig. S2); however, *Campylobacter* monocultures did not survive beyond 6–12 h without extracts from *S. aureus*. There were differences in the aerotolerance of *C. jejuni* and *C. coli* strains when incubated with cell-free extracts of *S. aureus* strains grown at different temperatures. For example, cells of *C. jejuni* NCTC11168 were still viable at the 12-h time point when incubated with media extracts from *S. aureus* B4-59C-25, B4-59C-37, B6-55A-4, B6-55A-25 and B6-55A-37 (Fig. 2E). *C. jejuni* OD2-67 cells were still viable after 12 h of aerobic incubation with extracts from B6-55A-37 (Fig. 2F), and *C. coli* WA3-33 cells were viable after 24 h of aerobic incubation with extracts from B6-55A-4 and B6-55A-25 (Fig. 2G). For *C. jejuni* T1-21, *C. coli* HC2-48 and ZV1-224, CFU counts were below the limit of detection (< 10^2^ CFU/ml) after 6 h of aerobic incubation in all used media. When statistical analysis was performed, effects of growth condition (monoculture vs polymicrobial culture), time, and time × growth condition interaction were all significant (*p* < 0.0001) on survival of *Campylobacter* in aerobic incubation at both temperatures (25 °C and 42 °C) (Supplementary Tables S2.4, S2.5).

### Biofilm formation

**Figure 3:**
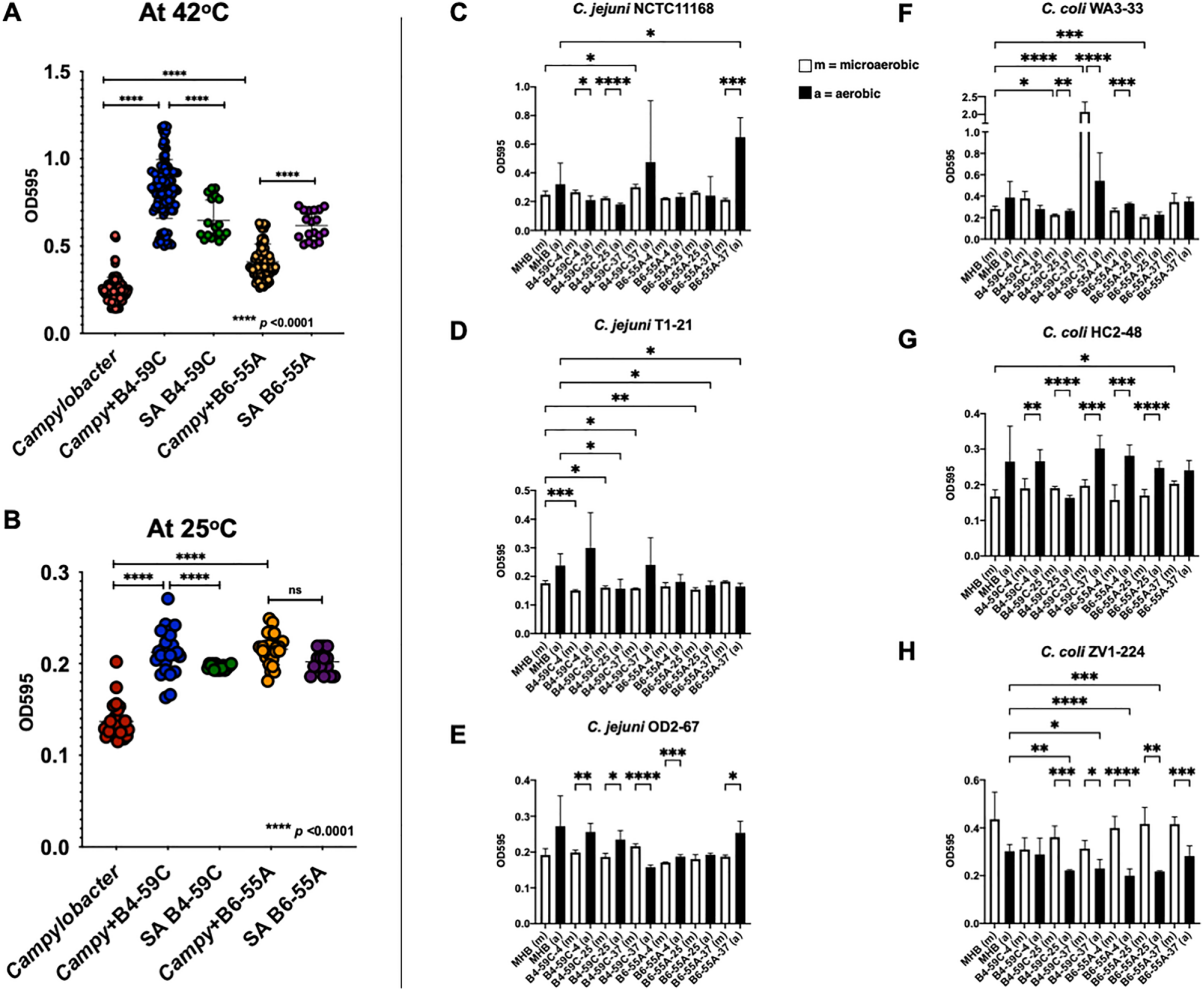
Biofilms produced by *Campylobacter* in mono and mixed cultures containing *S. aureus* strains or cell-free extracts. Biofilms adhering to polystyrene plates were measured at *A*_595_. (**A**,**B**) Biofilms formed by *Campylobacter* strains (all *C. jejuni/coli* strains) at 25 °C and 42 °C in mono and mixed cultures containing *S. aureus* strains B4-59C and B6-55A in aerobic condition. The production of biofilms by *S. aureus* B4-59C and B6-55A monocultures are included. (**C**–**H**) Biofilm formation of *Campylobacter* strains at 42 °C in cell-free filtrates from *S. aureus* media in microaerobic (m) and aerobic environment (a). Each bar in figures represents the mean value with SD error bar. Two-way ANOVA (Tukey multiple comparison tests) and Brown-Forsythe and Welch ANOVA tests (One-way ANOVA) were conducted in GraphPad Prism 9: *p < 0.05, **p < 0.01, ***p < 0.001, ****p < 0.0001.

Mixed populations of *Campylobacter* and *S. aureus* cells produced significantly larger biofilms than *Campylobacter* monocultures at both 25 °C and 42 °C (Fig. 3A,B, Supplementary Fig. S3). Biofilm formation was also greater with mixed populations of *Campylobacter* and *S. aureus* B4-59C than *S. aureus* monocultures at both temperatures. However, biofilm formation by *S. aureus* B6-55A monocultures was higher at 42 °C than with mixed cultures (Fig. 3B). Significant effect of growth condition (polymicrobial culture or monoculture) (p < 0.0001) was found at both temperatures (25 °C and 42 °C) on *Campylobacter* biofilm formation with *S. aureus* cells in two-way ANOVA analysis (Supplementary Table S2.7). Variable results were obtained when *Campylobacter* and cell-free extracts from *S. aureus* media were incubated in microaerobic and aerobic conditions at 42 °C (Fig. 3C–H). B4-59C-37 was able to enhance biofilm formation of *C. coli* WA3-33 and *C. jejuni* NCTC11168 strains in both microaerobic and aerobic condition when compared to control MHB. Higher biofilm formation was also found in B6-55A-37 for *C. jejuni* NCTC11168 (in aerobic environment) and *C. coli* HC2-48 (in microaerobic condition). Otherwise, most of the *Campylobacter* strains produced similar or lower amount of biofilm in cell free extracts from *S. aureus* media than control MHB in both aerobic and microaerobic environment (Fig. 3C–H). In MHB, all strains except *C. coli* ZV1-224 produced comparatively higher biofilm in aerobic condition than microaerobic condition. Significantly higher biofilm formation was seen in aerobic condition than microaerobic condition in most tested media except B4-59C-25 for *C. coli* HC2-48 (Fig. 3G) and likewise result for *C. jejuni* OD2-67 except in B4-59C-37 (Fig. 3E). Meanwhile, biofilm production was lower in aerobic condition than microaerobic condition for *C. coli* ZV1-224 in all tested media (Fig. 3H). Although none of the cell-free media significantly enhanced biofilm formation for all *Campylobacter* strains, growth medium factor (MHB control or cell free extract from *S. aureus* medium) had significant effect on *Campylobacter* biofilm formation in both aerobic and microaerobic incubation which was confirmed by statistical analysis with biofilm data from all *Campylobacter* strains (both *C. jejuni* and *C. coli* strains) (Supplementary Table S2.8). No consistent enhancement of biofilm production was observed when *Campylobacter* strains were incubated with the ≤ 30 kDa fraction of *S. aureus* media extracts (Supplementary Fig. S3C,D). However, biofilm results for *C. jejuni* OD2-67 in B4-59C-37 (≤ 30 kDa) was found to be significantly higher (*p* < 0.0001) among all tested media in microaerobic condition. The ≥ 30 kDa fraction could not be analyzed for its impact on biofilm production media due to the insignificant amount of concentrate obtained.

## Discussion

In most studies of foodborne pathogens, only a few targeted microorganisms are isolated and characterized^2,4^. However, polymicrobial colonization of retail meat products and meat processing environments is common^16,17,26^, and these polymicrobial conditions could have antagonistic or synergistic effects on foodborne pathogens^39^. *S. aureus* is ubiquitous and colonizes the skin, nose and hair of humans and other animals; consequently, the contamination of retail meat products with *S. aureus* strains occurs despite food safety measures. Most *S. aureus* strains in retail meat products originated from the respective animal^3,4,22,34^. Although the virulence of the *S. aureus* strains used in this study has not been tested, multiple virulence factors were identified in their genomes, including the gene encoding toxic shock syndrome^34^.

Prolonged survival of the six *Campylobacter* strains when cultivated with *S. aureus* cells or cell-free exudates indicates that *S. aureus* improves the survival of *Campylobacter* at lower temperatures. Previous studies regarding the transcriptional landscape of *Campylobacter* during survival at low temperature indicated that genes involved in quorum sensing and the acquisition of cryoprotectant molecules were differentially expressed^40–42^. Furthermore, *Campylobacter* was shown to utilize exogenous siderophores produced by other microorganisms^43,44^; these are used for iron acquisition during growth and survival and might also contribute to virulence. Our results showing improved survival of *Campylobacter* in *S. aureus* cell-free exudates indicates that metabolites produced by *S. aureus* improve the survival of *Campylobacter*. It is also important to note that *Campylobacter* cells undergo a transition to the VBNC stage during cold stress and remain viable for prolonged periods of time^14^. Hence, the presence of *S. aureus* cells or cell-free extracts might provide a protective environment for *Campylobacter* and facilitate viability for prolonged periods at lower temperatures. In this study, we counted culturable *Campylobacter* cells after incubation at 4 °C; however, this study did not address the impact of *S. aureus* on the VBNC condition of *Campylobacte*r cells. A previous report documented the detrimental effects of pre-established, organismal biofilms from poultry on the survival of *C. jejuni* at 10 °C^26^. Organisms identified in biofilms from poultry included *Pseudomonas* spp., *Staphylococcus* spp., *E. coli*, *Bacillus* spp., and *Flavobacterium* spp.^26^. During cold stress, a percentage of the *S. aureus* population undergoes a transition to a small colony variant (SCV), which showed alterations in amino acids as compared with normal cells^45^. It is also important to note that extracellular protein excretion by different *S. aureus* strains differs among strains, growth conditions and growth stage^46^. Various extracellular proteins including phosphoglycerate kinase, succinyl-CoA ligase, peroxiredoxin, superoxide dismutase, transmembrane sulfatase, and chaperonin were differentially expressed in *S. aureus* during growth at 37 °C at 12, 24 and 48 h^46^. In this study, SDS-PAGE analysis of *S. aureus* media extracts revealed changes in protein concentration and banding patterns at different pre-incubation temperatures (Supplementary Fig. S4, Supplementary Table S1). Further work is needed to determine the influence of amino acids and metabolites from *S. aureus* on *Campylobacter* survival, especially at lower temperatures.

The reduced levels of dissolved oxygen in mixed bacterial populations is another factor that contributes to the survival and growth of *Campylobacter*^47,48^*.* It is important to note that *S. aureus* can survive and multiply at refrigeration temperatures for prolonged periods of time^45,49^; this would lead to a consumption of available oxygen from media and the creation of an environment favorable for *Campylobacter*. Furthermore, *Campylobacter* was shown to tolerate aerobic conditions by metabolic commensalism with *Pseudomonas* spp.^50^, and a similar relationship might exist between *Campylobacter* spp. and *S. aureus*.

*Campylobacter* is a poor initiator of biofilm production in monoculture but is a well-established secondary colonizer of biofilms produced by other bacterial pathogens^26,51,52^. Variability in biofilm formation among *Campylobacter* strains in monoculture has been reported^27,29^; in general, *Campylobacter* biofilm formation is greater in aerobic than microaerobic conditions^30,38^. However, lower biofilm production in aerobic environment than microaerobic environment for some strains of *C. jejuni* has also been reported^53^. Nutrient-rich environments like chicken and liver juice were shown to enhance attachment, adhesion and biofilm formation by *Campylobacter* strains^18,19,29^. Significantly higher levels of biofilm were formed when *Campylobacter* strains were co-cultivated with *S. aureus* B4-59C at both 25 °C and 42 °C and suggested a mutually beneficial effect (Fig. 3A,B). In contrast, cumulative biofilm levels by *Campylobacter* and *S. aureus* B6-55A were significantly lower than biofilms produced by *S. aureus* monocultures at 42 °C (Fig. 3A). Similar interactions with mixed biofilm communities have been previously reported between *Campylobacter* and other organisms and may indicate antimicrobial activities or interspecies competition within the biofilm^27,54^. Meanwhile, previous studies reported better survival of *Campylobacter* strains in mixed biofilms during aerobic conditions than in monoculture^28,29^. Although flagella and *luxS*-mediated quorum sensing were suggested to be important for biofilm formation in *Campylobacter*^55^*,* the possibility of both flagellum-dependent and flagellum- independent biofilm mechanisms in *Campylobacter* has been suggested^38^. A previous study found no evidence for the role of interspecies cell signaling via autoinducer-2 among mixed populations, but instead suggested physical contact as the sole mechanism for biofilm formation when *Campylobacter* was a secondary colonizer^26^. Our results failed to find a specific, consistent influence for *S. aureus* growth media in *Campylobacter* biofilm formation, which may suggest that physical contact is needed to stimulate biofilm production in mixed populations. The ratio of the two bacterial pathogens might influence survival, aerotolerance and biofilm formation due to the availability of nutrients and quorum sensing. However, it is important to mention that the cell densities of *Campylobacter* and *S. aureus* would be far lower in retail meat environments as compared to the densities used in this study.

In this study, we show that *S. aureus* frequently occurs as a co-contaminant with *Campylobacter* in retail meat products. *S. aureus* enhances the survival of *C. jejuni* and *C. coli* strains at low temperatures and during aerobic conditions. Select strains of *S. aureus* potentially enhance biofilm formation by *Campylobacter* in aerobic conditions (normal atmospheric environment). Extracellular metabolites and proteins produced by *S. aureus* at multiple temperatures enhance the survival of *Campylobacter* strains at low temperature. The extracts produced by *S. aureus* in media improved the survival of some *Campylobacter* strains when compared to monocultures in MHB. However, *S. aureus* media extracts did not foster biofilm formation by *Campylobacter* strains in both aerobic and microaerobic environments.

In summary, it is well-established that the contamination of retail food products by *S. aureus* increases the risk of food poisoning. This study shows that contamination of retail meats by *S. aureus* also enhances the survival of *Campylobacter* during harsh environmental conditions. Hence, food safety measures are still needed to facilitate improved identification and reduced contamination of foodborne pathogens in mixed populations in retail meat and food products.

## Supplementary Information

Supplementary Information.
