# Supplementary material for: Staphylococcus aureus enhances biofilm formation, aerotolerance, and survival of Campylobacter strains isolated from retail meats
Source: Sci Rep. 2021 Jul 5;11:13837. doi: 10.1038/s41598-021-91743-w (PMC8257638; doi:10.1038/s41598-021-91743-w)
Supplement: Supplementary file 1 — Supplementary Information. [file 41598_2021_91743_MOESM1_ESM.docx]

Supplementary Data

***Staphylococcus aureus* enhances biofilm formation, aerotolerance, and survival of *Campylobacter* strains isolated from retail meats**

# Anand B. Karki^1^, Kaylee Ballard^1^, Claudia Harper^1^, Robert J. Sheaff^2^ and Mohamed K. Fakhr^1^*

1**Department of Biological Science, The University of Tulsa, Tulsa, Oklahoma, USA**

**^2^Department of Chemistry and Biochemistry, The University of Tulsa, Tulsa, Oklahoma, USA**

# *[mohamed-fakhr@utulsa.edu](mailto:mohamed-fakhr@utulsa.edu)

**Supplementary Tables**

**Table S1.** Protein concentration of cell free *S. aureus* grown media and Mueller Hinton Broth (MHB) (BCA assay, A562).

**Media (10 µl)** **Total protein (mg/ml) Media – MHB (mg/ml)**

MHB (Control) 0.681416 0

B6-55A-4 0.725664 0.044248

B6-55A-25 0.752212 0.070796

B6-55A-37 0.730088 0.048672

B4-59C-37 **0.867257 0.185841**

B4-59C-25 0.716814 0.035398

B4-59C-4 0.712389 0.030973

**Table S2.** Statistical analysis of survival and biofilm assays. ANOVA (One-way or Two-Way) were used for statistical analysis with survival and biofilm data in GraphPad Prism 9.

**Survival at 4^o^C**

**Table S2.1.** Two-way ANOVA analysis with repeated measure (Tukey multiple comparison test) for survival data for ***Campylobacter* strains** at 4^o^C in cell free *S. aureus* grown medium.

| **Effects** | **C. jejuni NCTC11168** | | | **C. jejuni T1-21** | | | **C. jejuni OD2-67** | | | **C. coli WA3-33** | | | **C. coli HC2-48** | | | **C. coli ZV1-224** | | |
| --- | --- | --- | --- | --- | --- | --- | --- | --- | --- | --- | --- | --- | --- | --- | --- | --- | --- | --- |
|  | **F^*^** | ***p* value** | | **F^*^** | ***p* value** | | **F^*^** | ***p* value** | | **F^*^** | ***p* value** | | **F^*^** | ***p* value** | | **F^*^** | ***p* value** | |
| **Time x Growth medium** | F (24, 56) = 4.832 | | <0.0001 | F (24, 56) = 1.366 | | 0.1682 | F (24, 56) = 3.073 | | 0.0003 | F (24, 56) = 5.081 | | <0.0001 | F (24, 56) = 6.270 | | <0.0001 | F (24, 56) = 8.792 | | <0.0001 |
| **Time** | F (1.184, 16.57) = 76.83 | | <0.0001 | F (1.002, 14.03) = 3.861 | | 0.0695 | F (1.000, 14.00) = 21.69 | | 0.0004 | F (1.031, 14.43) = 13.27 | | 0.0024 | F (1.000, 14.00) = 76.61 | | <0.0001 | F (1.000, 14.00) = 81.57 | | <0.0001 |
| **Growth medium** | F (6, 14) = 4.913 | | 0.0067 | F (6, 14) = 1.348 | | 0.3007 | F (6, 14) = 3.063 | | 0.0396 | F (6, 14) = 5.264 | | 0.005 | F (6, 14) = 6.229 | | 0.0023 | F (6, 14) = 8.887 | | 0.0004 |

***** F (DFn, DFd) = F (degrees of freedom in the numerator, degrees of freedom in the denominator)

All survival data (CFU counts) from different time points (0, 24, 48, 72, 120 and 168 hours) of each *Campylobacter* strains at 4^o^C in various growth media [ cell free *S. aureus* grown media (B4-59C-4, B4-59C-25, B4-59C-37, B6-55A-4, B6-55A-25, and B6-55A-37) and Mueller Hinton Broth (MHB)] were used for analysis. Effects of Time factor, growth medium and interaction of time and growth media were statistically significant (*p*<0.05) for all *Campylobacter* strains used in the study except *C. jejuni* T1-21.

**Table S2.2.** Two-way ANOVA analysis with repeated measure (Tukey multiple comparison test) for survival data of **all** ***Campylobacter* strains [ *C. jejuni* (n=3) and *C. coli* (n=3) strains]** at 4^o^C with *S. aureus* cells (total strains = 2).

| Effects | F (DFn, DFd) | p value |
| --- | --- | --- |
| Time x Growth condition (mono vs polymicrobial) | F (10, 255) = 0.7584 | 0.6688 |
| Time | F (1.898, 96.81) = 35.78 | <0.0001 |
| Growth condition (mono vs polymicrobial) | F (2, 51) = 2.107 | 0.1321 |
| Strains | F (51, 255) = 2.123 | <0.0001 |

All survival data [viable count (CFU)] of *Campylobacter* strains (all *C. jejuni* and *C. coli* strains) were analyzed by Two-way ANOVA with repeated measure for statistical significance of various effects. Growth condition [*Campylobacter* monoculture (mono) or *Campylobacter* cells with *S. aureus* cells (polymicrobial)] and Time x Growth condition interaction did not have significant effect (*p*>0.05) on survival of *Campylobacter* cells. Meanwhile, time effect and strain variability had significant effect on survivability of *Campylobacter* at 4^o^C in monoculture or polymicrobial growth condition. Viable counts were taken from time points 0, 24, 48, 72, 120 and 168 hours.

**Table S2.3.** Two-way ANOVA analysis with repeated measure (Tukey multiple comparison test) for survival data for ***Campylobacter* strains** at 4^o^C with *S. aureus* cells.

| Effects | C. jejuni NCTC11168 | | C. jejuni T1-21 | | C. jejuniOD2-67 | | C. coli WA3-33 | | C. coli HC2-48 | | C. coli ZV1-224 | |
| --- | --- | --- | --- | --- | --- | --- | --- | --- | --- | --- | --- | --- |
|  | F^*^ | *p* value | F^*^ | *p* value | F^*^ | *p* value | F^*^ | *p* value | F^*^ | *p* value | F^*^ | *p* value |
| Time x Growth condition | F (10, 30) = 20.33 | <0.0001 | F (10, 30) = 1.049 | 0.4293 | F (10, 30) = 19.77 | <0.0001 | F (10, 30) = 1.940 | 0.0786 | F (10, 30) = 3.707 | 0.0026 | F (10, 30) = 3.774 | 0.0023 |
| Time | F (1.730, 10.38) = 18.98 | 0.0005 | F (1.002, 6.013) = 17.73 | 0.0056 | F (1.078, 6.465) = 64.64 | 0.0001 | F (1.203, 7.220) = 29.07 | 0.0007 | F (1.908, 11.45) = 99.01 | <0.0001 | F (2.108, 12.65) = 29.52 | <0.0001 |
| Growth condition (mono vs polymicrobial) | F (2, 6) = 15.74 | 0.0041 | F (2, 6) = 0.9337 | 0.4436 | F (2, 6) = 23.88 | 0.0014 | F (2, 6) = 2.217 | 0.1902 | F (2, 6) = 20.01 | 0.0022 | F (2, 6) = 0.3105 | 0.7442 |

***** F (DFn, DFd) = F (degrees of freedom in the numerator, degrees of freedom in the denominator)

Statistical analysis for the effects of time factor, growth condition and their interaction mirrored the analysis used in **Table S2.2**. Time factor was significant for all *Campylobacter* strains. However, effect of growth condition was significant for *C. jejuni* strains NCTC11168, OD2-67 and *C. coli* strain HC2-48. Effect of time and growth condition interaction was significant for all strains except *C. jejuni* T1-21 and *C. coli* WA3-33.

**Aerotolerance assay**

**Table S2.4.** Two-way ANOVA with repeated measure analysis (Tukey multiple comparison test) for survival data of **all *Campylobacter* strains [*C. jejuni* and *C. coli* strains (total strains = 6)]** at 42^o^C in aerobic condition (aerotolerance assay) with *S. aureus* cells (total strains = 2).

| **Effects** | **F (DFn, DFd)** | **p value** |
| --- | --- | --- |
| **Time x Growth condition (mono vs polymicrobial)** | F (12, 165) = 39.10 | <0.0001 |
| **Time** | F (1.259, 69.23) = 101.8 | <0.0001 |
| **Growth condition (mono vs polymicrobial)** | F (4, 55) = 70.35 | <0.0001 |
| **Strains** | F (55, 165) = 1.007 | 0.4721 |

All survival data [(viable counts (CFU)] from different time points (0, 6, 12 and 24 hours) of *Campylobacter* strains in polymicrobial condition (with *S. aureus* cells) or monoculture (*Campylobacter* cells only) in aerobic conditions were used for analysis. Effects of time, growth condition and their interaction on survival of *Campylobacter* strains in aerobic condition at 42^o^C were found significant.

**Table S2.5.** Two-way ANOVA with repeated measure analysis (Tukey multiple comparison test) for survival data of **all *Campylobacter* strains (both *C. jejuni* and *C. coli* strains)** at 25^o^C in aerobic condition (aerotolerance assay) with *S. aureus* cells.

| **Effects** | **F (DFn, DFd)** | **p value** |
| --- | --- | --- |
| **Time x Growth condition (mono vs polymicrobial)** | F (12, 255) = 41.81 | <0.0001 |
| **Time** | F (3, 255) = 110.6 | <0.0001 |
| **Growth condition (mono vs polymicrobial)** | F (4, 85) = 81.60 | <0.0001 |
| **Strains** | F (85, 255) = 0.8930 | 0.7264 |

Statistical analysis mirrored that of **Table S2.4** and survival data (CFU counts) obtained from aerobic incubation at 25^o^C was analyzed. Effects of time, growth condition and their interaction on survival of *Campylobacter* strains in aerobic condition at 25^o^C were found significant.

**Table S2.6**. Two-way ANOVA analysis with repeated measure (Tukey multiple comparison test) for survival data for ***Campylobacter* strains** (only those strains which survived till 6 hours of aerobic incubation, **Figure 2E-G**) at 42^o^C with cell free *S. aureus* grown media in aerobic condition (aerotolerance assay).

| Effects | C. jejuni NCTC11168 | | C. jejuni OD2-67 | | C. coli WA3-33 | |
| --- | --- | --- | --- | --- | --- | --- |
|  | F (DFn, DFd) | *p* value | F (DFn, DFd) | *p* value | F (DFn, DFd) | *p* value |
| Time x Growth medium | F (12, 28) = 2.208 | 0.0413 | F (12, 28) = 1.033 | 0.4475 | F (12, 28) = 1.049 | 0.4355 |
| Time | F (1.067, 14.93) = 17.30 | 0.0007 | F (1.000, 14.00) = 25.37 | 0.0002 | F (1.006, 14.08) = 5.477 | 0.0343 |
| Growth medium | F (6, 14) = 1.798 | 0.1715 | F (6, 14) = 1.034 | 0.444 | F (6, 14) = 0.9219 | 0.5083 |

Only three *Campylobacter* strains (*C. jejuni* NCTC11168, *C. jejuni* OD2-67 and *C. coli* WA3-33) could survive till 6 hours of aerobic incubation at 42^o^C in tested media [cell free *S. aureus* grown media (B4-59C-4, B4-59C-25, B4-59C-37, B6-55A-4, B6-55A-25, and B6-55A-37) and MHB]. Survival data (CFU counts) of *Campylobacter* strains (*C. jejuni* strains (0, 6 and 12 hours), *C. coli* strain (0, 12 and 24 hours) were analyzed. Only time factor had significant effect on survival in aerobic incubation for all strains (*p*<0.05).

**Biofilm assay**

**Table S2.7.** Two-Way ANOVA analysis (Tukey multiple comparison test) with the data of biofilm of **all *Campylobacter* strains [all *C. jejuni* and *C. coli* strains (total strains = 6)]** with *S. aureus* cells at 42^o^C and 25^o^C.

| **Effect** | **at 42^o^C** | | **at 25^o^C** | |
| --- | --- | --- | --- | --- |
|  | **F (DFn, DFd)** | ***p* value** | **F (DFn, DFd)** | ***p* value** |
| **Growth condition (Mono vs polymicrobial)** | F (4, 248) = 309.8 | <0.0001 | F (4, 76) = 70.54 | <0.0001 |

Biofilm data of *Campylobacter* strains in monoculture or polymicrobial condition [with *S. aureus* cells (*S. aureus* B4-59C and B6-55A)] at 42^o^C and 25^o^C were analyzed. Effect of growth condition on biofilm was found significant for both 42^o^C and 25^o^C.

**Table S2.8.** Two-Way ANOVA analysis (Tukey multiple comparison test) with the data of Biofilm of ***Campylobacter* strains (all *C. jejuni* and *C. coli* strains)** with cell free *S. aureus* grown medium 42^o^C in microaerobic and aerobic incubation.

| Effect | Microaerobic | | Aerobic | |
| --- | --- | --- | --- | --- |
|  | F (DFn, DFd) | *p* value | F (DFn, DFd) | *p* value |
| Growth medium | F (6, 318) = 10.48 | P<0.0001 | F (6, 324) = 10.12 | <0.0001 |

Biofilm data of *Campylobacter* strains in cell free *S. aureus* grown media at 42^o^C in microaerobic or aerobic condition were analyzed. Effect of growth medium on biofilm formation in both microaerobic and aerobic incubation was significant.

**Table S3.** Cell counts of *S. aureus* B4-59C and B6-55A at 4^o^C in mixed cultures with *C. jejuni* (T1-21, OD2-67, NCTC11168) and *C. coli* (WA3-33, HC2-48, ZV1-224). Viable counts of *S. aureus* were obtained on mannitol salt agar, and values represent log CFU/ml ± standard error.

| **Mixed culture** | **0 hour** | **24 hours** | **48 hours** | **72 hours** | **120 hours** | **168 hours** |
| --- | --- | --- | --- | --- | --- | --- |
| **T1-21+B4-59C** | 6.865±0.051 | 7.114±0.12 | 6.426±0.038 | 6±0.088 | 5.959±0.138 | 5.734±0.128 |
| **OD2-67+B4-59C** | 6.699±0.051 | 6.865±0.167 | 6.669±0.142 | 5.609±0.013 | 5.656±0.181 | 5.254±0.203 |
| **11168+B4-59C** | 6.702±0.183 | 6.885±0.066 | 6.114±0.087 | 6.627±0.103 | 6.195±0.076 | 5.091±0.134 |
| **WA3-33+B4-59C** | 6.637±0.179 | 6.954±0.265 | 6.239±0.107 | 6.653±0.024 | 6.154±0.175 | 5.669±0.065 |
| **HC2-48+B4-59C** | 6.903±0.079 | 7±0.141 | 6.349±0.08 | 6.518±0.046 | 6.156±0.026 | 5.587±0.128 |
| **ZV1-224+B4-59C** | 6.637±0.087 | 7.103±0.12 | 5.802±0.164 | 6.491±0.027 | 5.738±0.07 | 5.576±0.082 |
| **T1-21+B6-55A** | 6.753±0.202 | 6.669±0.108 | 6.591±0.089 | 6.156±0.2 | 5.329±0.174 | 6.068±0.103 |
| **OD2-67+B6-55A** | 6.802±0.059 | 6.896±0.149 | 6.591±0.083 | 6.156±0.115 | 5.663±0.038 | 5.873±0.103 |
| **11168+B6-55A** | 6.527±0.039 | 6.743±0.163 | 6.349±0.025 | 6.404±0.012 | 5.556±0.116 | 5.678±0.052 |
| **WA3-33+B6-55A** | 6.845±0.109 | 6.637±0.157 | 6.58±0.007 | 6.271±0.093 | 5.286±0.110 | 6.125±0.075 |
| **HC2-48+B6-55A** | 6.637±0.032 | 6.824±0.06 | 5.985±0.147 | 6.263±0.086 | 6.197±0.111 | 5.678±0.079 |
| **ZV1-224+B6-55A** | 6.518±0.044 | 6.885±0.066 | 5.845±0.036 | 6.308±0.063 | 5.998±0.104 | 5.616±0.061 |

# Supplementary Figures

**Figure S1**. Survival of *Campylobacter strains* in mono and mixed cultures containing *S. aureus* strains B4-59C and B6-55A at 4^o^C. Each bar represents mean value of Log CFU/ml with SD error bars. Horizontal dotted lines in the figures represent the limit of detection (LOD, ~33 CFU/ml) of CFU counts in the assays. Bars in the figures not exceeding the LOD line represent the readings with no detectable CFU counts at each respective time point. (Statistical analysis [Tukey multiple comparison test (Two-way ANOVA with repeated measures)] in GraphPad Prism 9: * *p* <0.05, ***p* <0.01, ****p* <0.001, *****p* <0.0001.

**Figure S2**. Survival of *Campylobacter* strains (all *C. jejuni* and *C. coli* strains) in mono and mixed cultures containing *S. aureus* strains B4-59C and B6-55A in aerobic incubation at **(A)** 25^o^C and **(B)** 42^o^C. All individual readings of CFU counts of all *Campylobacter* strains and *S. aureus* strains from monocultures and mixed cultures are represented in the figures. Horizontal dotted lines in the figures represent the limit of detection (LOD, ~ 33 CFU/ml) for CFU counts in these assays. All data points plotted on the dotted line (LOD) represent the readings with no detectable CFU counts for the respective time points. Error bars represent the SD values. Statistical analysis [Tukey multiple comparison test (Two-way ANOVA with repeated measures)] was conducted in GraphPad Prism 9: * *p* <0.05, ***p* <0.01, ****p* <0.001, *****p* <0.0001.

**
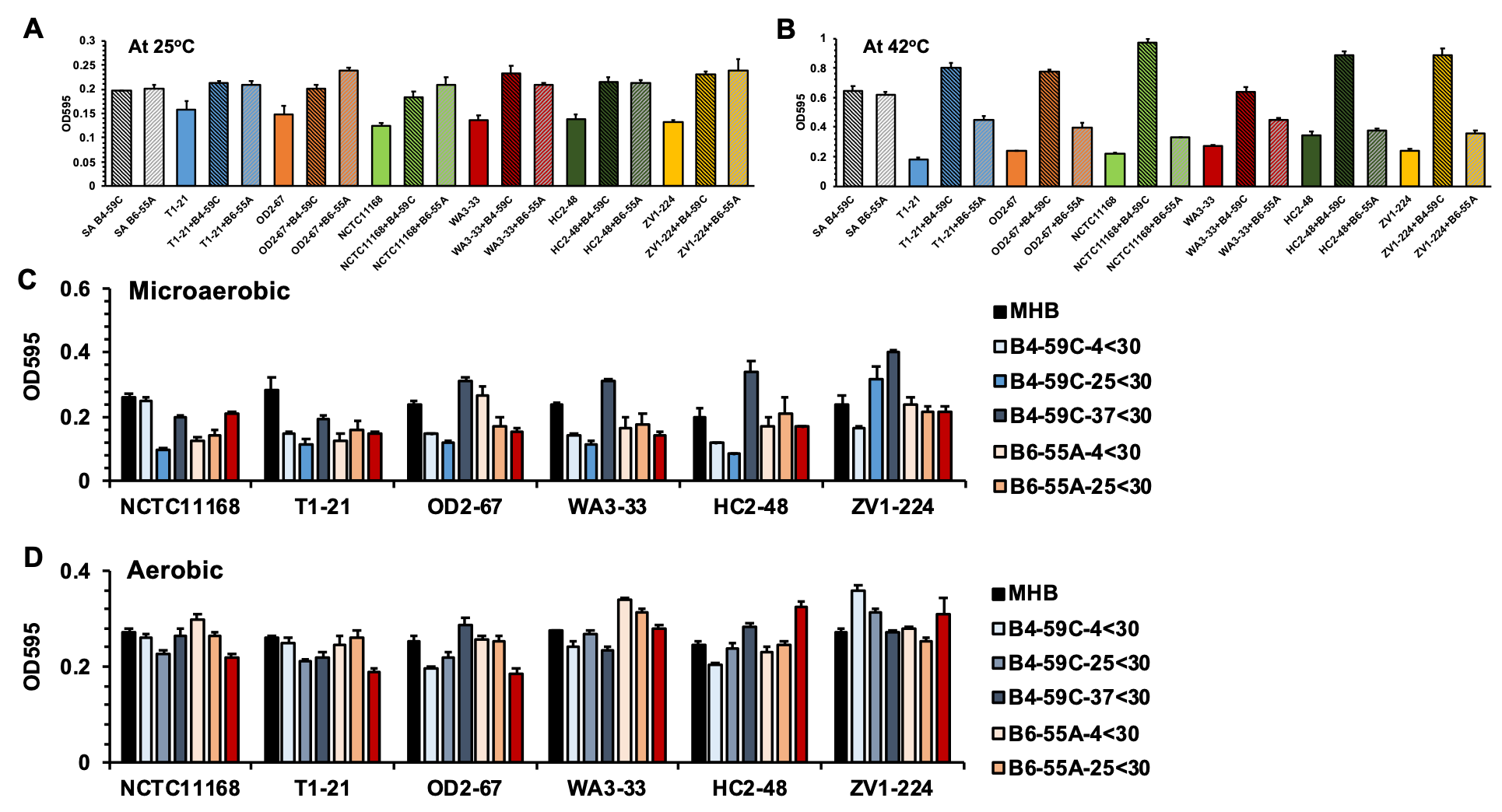
**

**Figure S3.** Biofilm formation of *S. aureus* B4-59C and B6-55A, *C. jejuni* T1-21, OD2-67, and NCTC11168, and *C. coli* WA3-33, HC2-48, and ZV1-224 with *S. aureus* cells. Cells were incubated as mono and mixed cultures at **(A)** 25^o^C and **(B)** 42^o^C in aerobic condition and analyzed for biofilm production. Biofilm assay of *Campylobacter* strains incubated in <30 kDa fraction of cell free *S. aureus* grown media and MHB (controls) (**Fig. C, D**), cells were incubated under **(C)** aerobic and **(D)** microaerobic conditions at 42^o^C. Each bar in figures represent mean value of absorbance readings with SD error bars.


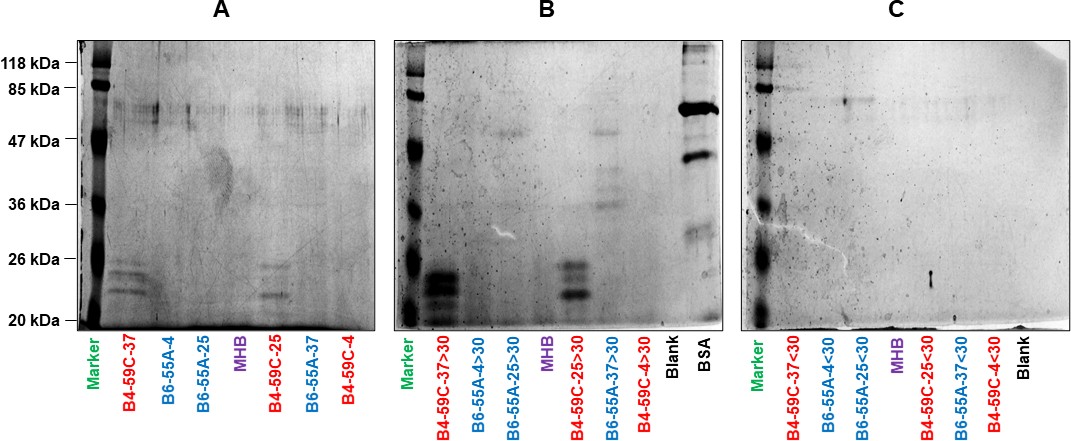


**Figure S4.** SDS-PAGE gel of proteins in *S. aureus* media extracts. (**A**) Cell-free *S. aureus* media extracts. (**B**) Proteins in ≥30 kDa fraction from *S. aureus* media extracts. (**C**) Proteins in ≤30 kDa flow-through fraction of *S. aureus* media extracts. Twenty microliter sample volumes were loaded into wells and analyzed by 12% SDS-PAGE.
